# Effect of Sodium-Glucose Co-transporter Protein 2 Inhibitors on Arrhythmia in Heart Failure Patients With or Without Type 2 Diabetes: A Meta-Analysis of Randomized Controlled Trials

**DOI:** 10.3389/fcvm.2022.902923

**Published:** 2022-05-18

**Authors:** Ziwei Yin, Huizhen Zheng, Zhihua Guo

**Affiliations:** ^1^Department of Cardiology, The First Affiliated Hospital of Hunan University of Chinese Medicine, Changsha, China; ^2^College of Chinese Medicine, Hunan University of Chinese Medicine, Changsha, China

**Keywords:** sodium-glucose co-transporter type 2 inhibitors, type 2 diabetes mellitus, heart failure, arrhythmia, atrial fibrillation, meta-analysis

## Abstract

**Aim:**

Arrhythmic events such as atrial fibrillation (AF) are tightly associated with an increased risk of heart failure (HF). Previous studies have shown inconsistent results regarding the association between sodium-glucose co-transporter 2 inhibitors (SGLT2i) and the risk of arrhythmia. The purpose of this study was to investigate the association of SGLT2i treatment with arrhythmia outcomes in clinical trials of patients with HF.

**Methods:**

We searched Embase, PubMed, Web of Science, Medline, The Cochrane Library, and JAMA databases to identify appropriate randomized controlled trials (RCTs) of SGLT2i interventions. Endpoint outcomes included AF, atrial flutter (AFL), AF/AFL, ventricular fibrillation (VF), ventricular tachycardia (VT), VF/VT, and bradycardia. A random-effects model was used for the meta-analysis of all outcomes. The risk of bias and quality of evidence was assessed by using the Cochrane tool and assessment framework.

**Results:**

Out of 1,725 citations, 9 trials were included in this study, with follow-up from 4 weeks to 52 weeks for 10,344 participants (mean age 68.27 years; 69.62% of participants were men). Compared with placebo, SGLT2i reduced the incidence of AF by 37% [ratio risk (RR) 0.63; 95% confidence interval (CI) 0.45–0.87; *p* < 0.05] and AF/AFL by 34% (RR 0.66; 95% CI 0.49–0.90; *p* < 0.05).

**Conclusions:**

SGLT2i can reduce the risk of cardiac arrhythmias, particularly the AF. Our study provides strong evidence for recommending the use of SGLT2i in patients with HF.

**Systematic Review Registration:**

PROSPERO, identifier: CRD42022296696.

## Introduction

Arrhythmic events are one of the major risk factors for heart failure (HF), and approximately 25% of HF patients have atrial fibrillation (AF) caused by mechanisms such as atrial pressure overload and enlargement, altered myocardial conduction, dysregulated gene expression, and structural remodeling (1). Meanwhile, HF also induces AF due to the common pathophysiological mechanisms and risk factors (2). Therefore, it is important to reduce the risk of cardiac arrhythmias in patients with HF. However, the pharmacological treatment for arrhythmia is limited, and the majority of antiarrhythmic drugs have significant side effects or toxicities (3). Lately, sodium-glucose co-transporter 2 inhibitors (SGLT2i) have attracted remarkable attention because of its widespread use in cardiovascular disease.

SGLT2i is a new class of anti-diabetic medications, which reduce glucose reabsorption by combining with SGLT2i in the luminal membrane of the early proximal tubule. Apart from the hypoglycemic effects, the cardiovascular benefits of SGLT2i have been demonstrated in numerous randomized clinical trials (RCTs) (4–7). A secondary analysis from the EMPEROR-Reduced trial showed that empagliflozin significantly reduced the risk of cardiovascular death or hospitalization for heart failure (HHF) (8). However, the arrhythmic benefit of SGLT2i in HF patients is controversial despite its extraordinary cardiovascular benefit. A growing number of studies have proved the benefit of SGLT2i in AF (9–13). On the contrary, many studies have come to diametrically opposite conclusions (14, 15). Hitherto, there have been no meta-analyses to investigate the arrhythmia benefits of SGLT2i in HF patients. Therefore, it is necessary to confirm the arrhythmia benefits of SGLT2i to HF patients with or without type 2 diabetes mellitus (T2DM) by meta-analysis.

Currently, there is considerable controversy regarding the arrhythmic benefits of SGLT2i, and no studies have been conducted on the independent group of HF participants. Therefore, our research aimed at analyzing whether SGLT2i is beneficial to arrhythmia in HF patients, and providing new recommendations to clinicians in treating arrhythmia.

## Methods

This meta-analysis is reported according to the Preferred Reporting Items for Meta-Analyses (PRISMA) statement (Appendix 1), and we registered it in the PROSPERO database (CRD42022296696).

### Study Selection and Inclusion Criteria

Two investigators (ZH and YZ) independently screened records and negotiated disagreements. We included trials that met the following criteria: (1) randomized controlled trials (RCTs); (2) adults with a confirmed diagnosis of HF with or without T2DM; and (3) SGLT2i vs. placebo as the intervention. The outcomes of interest include AF, AFL, AF/AFL, VT, VF, VT/VF, and bradycardia. Observational studies and studies with a follow-up duration <1 month were excluded. If replicated studies of the same trial provided similar results, we included only the published studies with the most comprehensive data needed.

**Figure 1:**
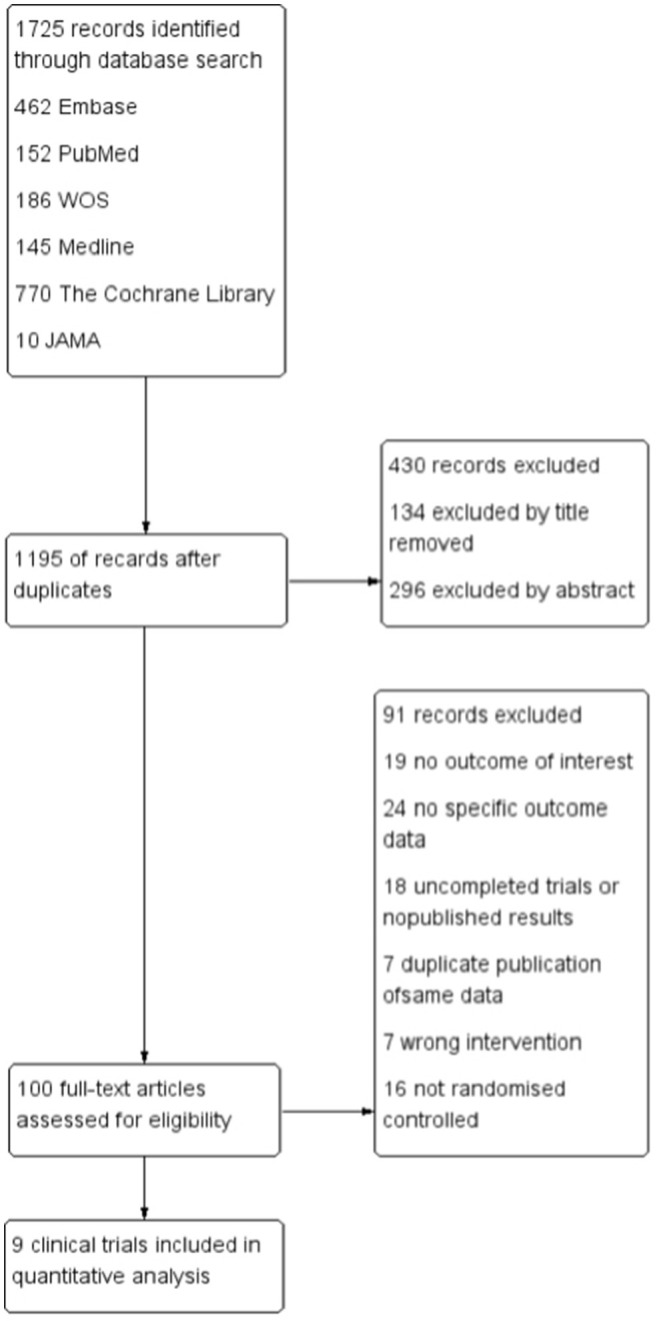
Flow diagram of literature search and study selection.

All articles searched in the database were imported into EndNote X9. Two researchers independently selected eligible studies by screening titles and abstracts and evaluating the full text, cross-checking for compliance with the criteria by two researchers for studies that needed to be included, and recording the reasons for exclusion for studies that were excluded, with disagreements resolved by consensus. Figure 1 provides the complete study selection process.

### Search Strategy and Selection Criteria

We searched Embase, PubMed, Web of Science, Medline, The Cochrane Library, and JAMA databases for studies published from the time of their creation to 25 February 2022. In addition, we checked trial records submitted to ClinicalTrials.gov for additional reports on HF patients from any SGLT2i trials. We considered all studies potentially eligible for review, while manually searching for references to relevant reviews and articles without language restrictions. We used the following combined text and MeSH terms: “sodium-glucose co-transporter type 2 inhibitors,” “heart failure,” and “randomized controlled trial.” We have improved the search formula according to the PICOS principle, which has been added in Appendix 2.

### Data Extraction and Quality Assessment

Two investigators extracted data independently for each included study using a standardized data extraction form. Information extracted included: basic information about the study, participant characteristics, and interventions.

We used the Cochrane Risk of Bias Assessment Tool to assess trials for selection bias, performance bias, detection bias, attrition bias, reporting bias, and other biases. Each study was categorized as low risk (green), unclear risk (yellow), or high risk (red) of bias. The certainty of the evidence was assessed using the Grading of Recommendations Assessment, Development, and Evaluation (GRADE) assessment.

### Statistical Analysis

The primary outcome indicator of this study was the number of event-specific individuals, so we calculated ratio risk (RR) and 95% confidence interval (95% CI) as dichotomous variable analysis statistics and combined them using a random-effects model with statistical significance set at 0.05. Subgroup analyses were performed according to the type of HF and the SGLT2i drug used. In addition, we assessed agreement between included studies and assessed heterogeneity using the *I*^2^ statistics, with no significant heterogeneity considered when the threshold *p* > 0.10 and *I*^2^ < 50 % and significant heterogeneity considered if the threshold *p* < 0.10 or *I*^2^ > 50%, using subgroup analysis or meta-regression to investigate potential sources of heterogeneity. Asymmetries in the funnel plots determined by visual assessment or a *p*-value for Egger's test < 0.10 would suggest potential publication bias. Differences were considered statistically significant at *p* < 0.05. All data analyses were performed using Review Manager (RevMan) 5. Version 3 software (The Cochrane Collaboration, Copenhagen, Denmark).

## Results

Among the 1,725 citations identified by the literature search, 1,195 records were removed because of duplicates, and another 430 were excluded according to the titles and abstracts. Of the 100 studies identified, 91 were removed after reading the complete article. Finally, 9 studies met our inclusion criteria and included 10,344 participants (Figure 1) (16–23). Symmetry was observed in the funnel plots for each outcome, which was shown in Appendix 3. Egger's test for each outcome did not reveal significant asymmetry, which was shown in Appendix 4. The result of the GRADE assessment for each outcome was shown in Appendix 5.

### Baseline Characteristics of the Included Studies

Table 1 summarizes the main characteristics of the 9 RCTs (10,344 patients) included. These studies were published between 2020 and 2021. The mean age of the participants was 68.27 years; 69.62% (*n* = 7,202) of them were male, and 30.38% (*n* = 3,142) were female. The longest follow-up duration was 52 weeks in the EMPEROR-Reduced trial, and the shortest follow-up duration was 4 weeks in the EMPA-RESPONSE-AHF trial.

**Table 1:** Main characteristics of the 10 randomized controlled trials in this meta-analysis.

**Trial/Year**	**Country/area**	**Participants**	**Mean age**	**Overall sample size**	**T2DM**	**Interventions**		**Follow-up period**	**No. of participants**		**Male%**
Determine-reduced, 2021	Multiple center	HFrEF	67.8	313	With or without	Placebo	Dapagliflozin	16 weeks	157	156	74.4%
Determine-preserved, 2021	Multiple center	HFpEF	71.8	504	With or without	Placebo	Dapagliflozin	16 weeks	251	253	63.5%
Empa-tropism, 2021	United States	HF	62	84	Without	Placebo	Empagliflozin	6 months	42	42	64.3%
Emperial-reduced, 2020	United States	HFrEF	69.0	312	With or without	Placebo	Empagliflozin	12 weeks	156	156	74.4%
Emperial-preserved, 2020	United States	HfpEF	73.5	315	With or without	Placebo	Empagliflozin	12 weeks	158	157	56.8%
Empa-response-Ahf, 2020	Multiple center	HF	76.0	79	With or without	Placebo	Empagliflozin	4 weeks	39	40	67.1%
Emperor-reduced, 2021	Multiple center	HFrEF	66.8	3,730	With or without	Placebo	Empagliflozin	52 weeks	1,867	1,863	76.1%
Dapa-Hf, 2020	Multiple center	HFrEF	66.3	4,744	With or without	Placebo	Dapagliflozin	8 months	2,373	2,371	76.6%
Define-Hf, 2021	United States	HFrEF	61.3	263	With or without	Placebo	Dapagliflozin	12 weeks	132	131	73.4%

*HF, heart failure; HFrEF, heart failure with reduced ejection fraction; HFpEF, heart failure with preserved ejection fraction; SGLT2i, sodium-glucose cotransporter protein 2 inhibitors; T2DM, type 2 diabetes mellitus*.

### Risk of Bias and Quality of Evidence

All included studies were described as randomized, blinded, and blinding was done primarily through the Interactive Voice/Web Response System. Nine studies were registered on ClinicalTrials.gov and identified with a National Clinical Trial number. Nonetheless, AF or any arrhythmia event was recorded as a serious adverse event rather than a primary outcome, posing an unknown risk in selective reporting. Based on a review of their protocols, these studies were considered to have a low risk of bias in selective reporting, whereas other trials that did not report sufficient information had an unclear risk of bias in this area. In addition, seven trials were funded by pharmaceutical companies, so there was an uncertain risk of other biases. Appendix 6 depicts the results of the Cochrane risk of bias assessment.

### Outcomes of Atrial Fibrillation

**Figure 2:**
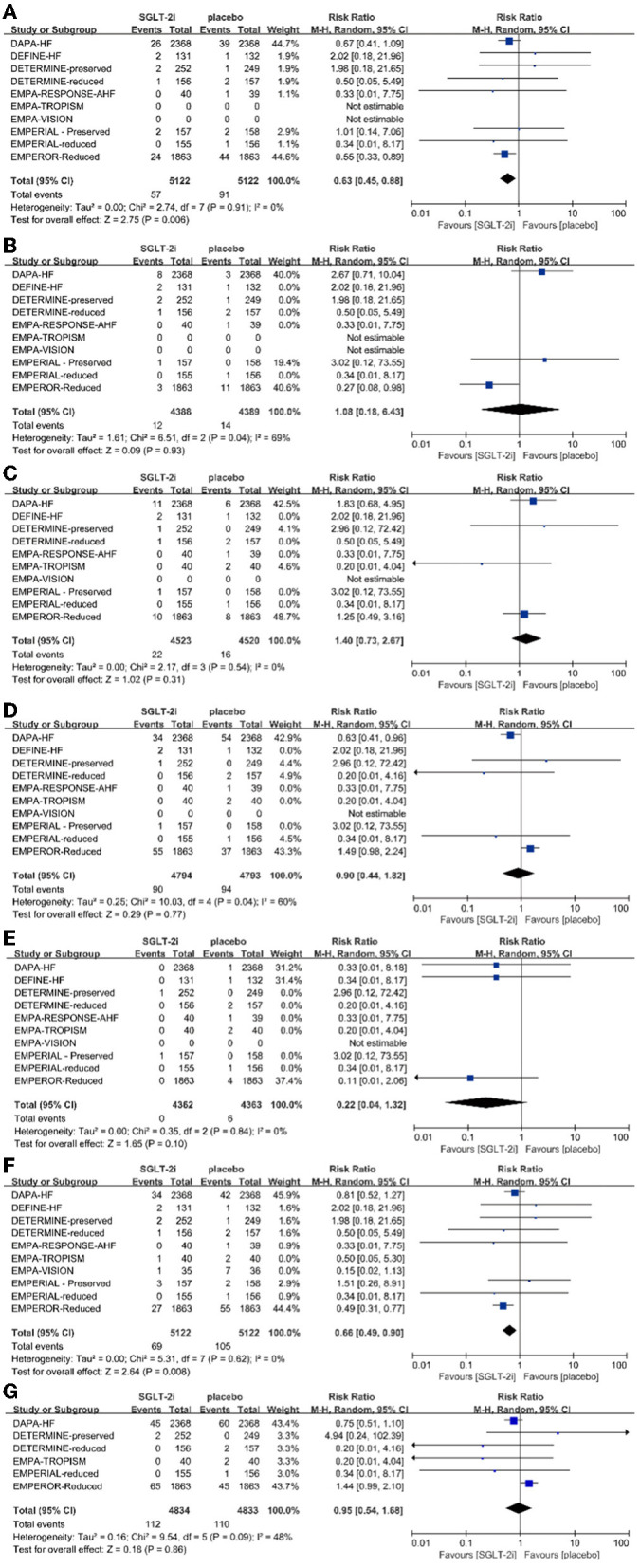
Effect of SGLT2 inhibitors compared with placebo on **(A)** atrial fibrillation, **(B)** atrial flutter, **(C)** ventricular fibrillation, **(D)** ventricular tachycardia, **(E)** bradycardia, **(F)** AF/AFT, and **(G)** VF/VT.

A total of 10,260 patients reported 148 cases of AF in 8 trials. Of the 5,122 patients treated with SGLT2i, 57 AF events were observed, and 91 AF events were observed among 5,122 patients in the placebo group. Overall, SGLT2i reduced the odds of AF by 37% (RR 0.63; 95% CI 0.45–0.88; *p* < 0.05) compared with placebo (Figure 2), with no significant heterogeneity between trials (*p* = 0.91, *I*^2^ = 0%). There was no significant heterogeneity between subgroups according to the type of HF (*p* = 0.31) and SGLT2i (*p* = 0.22) (Appendix 7).

### Outcomes of Atrial Flutter

In the 3 RCTs, a total of 8,789 patients with a median follow-up duration of 32 weeks reported 26 cases of AFL. Twelve AFL events were observed in 4,388 patients treated with SGLT2i, and 14 AFL events among 4,398 patients in the placebo group were observed. SGLT2i did not significantly affect the risk of AFL compared with placebo (RR 1.08; 95% CI 0.18–6.43; *p* = 0.93) (Figure 2). There was a large heterogeneity between trials (*p* = 0.04, *I*^2^ = 69%). When AF and AFL are combined as a composite endpoint, the risk of AF/AFL was reduced by 34% (RR 0.66; 95% CI 0.49–0.90; *p* = 0.008) (Figure 2). There was no significant heterogeneity between trials (*p* = 0.62; *I*^2^ = 0%).

### Outcomes of Ventricular Fibrillation

A total of 9,062 patients in 4 RCTs with a median follow-up duration of 31 weeks reported 38 cases of VF. Among 4,523 patients treated with SGLT2i, 22 cases of VF were observed, and 16 cases of VF were observed among 4,520 patients in the placebo group. SGLT2i did not significantly affect the risk of VF compared with placebo (RR 1.40; 95% CI 0.73–2.67; *p* = 0.31) (Figure 2). There was no significant heterogeneity (*p* = 0.54; *I*^2^ = 0%).

### Outcomes of Ventricular Tachycardia

A total of 9,003 patients with a median follow-up duration of 25.6 weeks reported 184 cases of VT in 5 RCTs. Among 4,794 patients treated with SGLT2i, 90 VT events were observed, and 94 VT events were observed among 4,793 patients in the placebo group. SGLT2i did not significantly affect the risk of VT compared with placebo (RR 0.90; 95% CI 0.44–1.82; *p* = 0.77) (Figure 2), which was not significant. There was heterogeneity between trials (*p* = 0.04; *I*^2^ = 60%).

### Outcomes of Bradycardia

In 3 RCTs with a total of 8,737 patients and a median follow-up duration of 32 weeks, 6 cases of bradycardia were reported. No bradycardia events were observed among the 4,362 patients treated with SGLT2i, and 6 bradycardia events were observed among the 4,363 patients in the placebo group. SGLT2i did not significantly affect the risk of bradycardia compared with placebo (RR 0.22; 95% CI 0.04–1.32; *p* = 0.10) (Figure 2). There was no significant heterogeneity between trials (*p* = 0.84; *I*^2^ = 0%).

### Outcomes of VF/VT

In 6 RCTs with a total of 9,667 patients and a median follow-up duration of 25 weeks, 222 cases of VF/VT were reported. Among 4,834 patients treated with SGLT2i, 112 VF/VT events were observed, and 110 VF/VT events were observed among 4,833 patients in the placebo group. SGLT2i did not significantly affect the risk of VF/VT compared with placebo (RR 0.95; 95% CI 0.54–1.68; *p* = 0.86) (Figure 2). There was no significant heterogeneity between trials (*p* = 0.09; *I*^2^ = 48%).

## Discussion

In this systematic review and meta-analysis of 9 RCTs, 10,344 HF patients with or without T2DM were ultimately included. To the authors' knowledge, this is the largest and most comprehensive systematic review and meta-analysis that investigated the association between SGLT2i and arrhythmic events in patients with HF. We found that SGLT2i was related to a 37% lower risk of AF and a 34% lower risk of AF/AFL compared with placebo, which had nothing to do with different types of drugs or diseases. In contrast, no significant reduction was found in the incidence of VF, VFT, VF/VFT, and bradycardia.

Several recent studies have investigated whether SGLT2i has benefits in AF. Specific data is shown in Table 2. Some meta-analyses have found that SGLT2i reduces the risk of atrial fibrillation by approximately 19% (9–12). However, participants in their study were not 100% HF patients and included patients with chronic kidney disease or other cardiovascular diseases (12, 13). Therefore, it is not clear what impact SGLT2i has on HF patients. To provide more clarity on the role of SGLT2i in HF patients, we set the diagnosed HF patients as an inclusion criterion to exclude confounders from any other aspects, so the correlation we identified was more reliable. Our research has come up with an even more surprising result: SGLT2i was associated with a 37% reduction in the risk of atrial fibrillation events in 100% of participants with heart failure, regardless of whether these patients had diabetes or not.

**Table 2:** Differences between our study and other previous studies.

**References**	**Type**	**Condition**	**HF%**	**Reduced risk of AF or AFL**
This study, 2022	Meta-analysis	HF	100%	AF 37%, AF/AFL 34%
Zelniker et al. (9)	RCT	T2DM, high risk for CVD	NA	AF/AFL 19%
Li et al. (11)	Meta-analysis	HF, T2DM, CKD	21.4%	AF 18%
Fernandes et al. (12)	Meta-analysis	HF or T2DM	NA	AF 19%
Butt et al. (14)	RCT	HF, T2DM	100.0%	No significant
Usman et al. (15)	Meta-analysis	CVD, T2DM	NA	No significant
Okunrintemi et al. (13)	Meta-analysis	HF, T2DM, CKD, CVD	NA	AF 21%

*HF, heart failure; T2DM, type 2 diabetes mellitus; AF, atrial fibrillation; AFL, atrial flutter; NA, not applicable; CKD, chronic kidney disease; CVD, cardiovascular disease; RCT, randomized controlled trial*.

As the potential benefits of SGLT2i for arrhythmic events are increasingly supported by much research, its intrinsic pathological mechanisms have become a hotly debated topic. As we all know, SGLT2 focuses on the kidney rather than the heart (24, 25). However, a large number of studies have shown that SGLT2i can reduce the incidence of AF through many other pathways. Mitochondrial abnormality is the characteristic feature of atrial specimens in AF patients (26), which may be an important factor for AF induction in HF patients. Mitochondria are widely distributed in the myocardium and are important organelles for maintaining the function of cardiomyocytes (27). Adenosine triphosphate (ATP), a high-energy molecule synthesized in mitochondria, provides a large amount of energy for the myocardium (28). However, this process is usually accompanied by the production of reactive oxygen species (ROS), which are cardiotoxic byproducts (29). ROS is excessively increased when mitochondrial function is impaired (30), especially in diabetic heart disease (31). On the one hand, SGLT2i can increase the expression of peroxisome proliferator-activated receptor γ coactivator-1 alpha (PGC-1α) (32, 33), and ensure the normal physiological function of the mitochondria (33, 34). PGC-1α is an important regulator of mitochondrial biosynthesis, and the decrease in the PGC-1 level is considered to be the cause of mitochondrial dysfunction (35). On the other hand, canagliflozin can increase the level of β-hydroxybutyric acid (β-HA) and acetic acid in the AF canine model (36). Ketones, as a more efficient super fuel than free fatty acids (37), cause more ATP production in cardiomyocytes to resist mitochondrial damage (38, 39) and exhibit anti-arrhythmia potential by stabilizing cell membrane potential (40). Higher levels of β-HA also reduced the cardiotoxicity associated with excessive ROS (41). At the same time, atrial substrates are driven by an abnormal rise in ROS, leading to a sustained rise in late Na+ currents (42), which is characteristic of cardiomyocytes in HF patients (43). It has been shown that excessive Na^+^ levels promote the development of AF (44, 45), and Na^+^ homeostasis is tightly linked to Ca^2+^ homeostasis through ion channels such as the Na^+^/Ca^2+^ exchanger (NCX) (46, 47) and SGLT2i can act on Na^+^/H^+^ exchanger 1 (NHE1) to significantly reduce Na^+^ and Ca^2+^ in the myocardial cytoplasm (48–50).

In addition, SGLT1 and SGLT2 are both members of the solute-carrier family-5 (SLC5) (25). Different from SGLT2, SGLT1 is widely distributed in the human heart (51–54). Despite the high selectivity of SGLT2i for SGLT2 (55), we cannot help wondering whether the cardiac benefits of SGLT2i are related to the wide intracardiac distribution of SGLT1. SGLT1 showed a high affinity for empagliflozin in a molecular docking study (56). The high expression of SGLT1 is associated with the increased NADPH oxidase-related ROS levels and the expression of pro-inflammatory genes, while, canagliflozin can alleviate oxidative stress and inflammation by inhibiting SGLT1 (57). A recent study found that inhibiting SGLT1 could prevent fatal arrhythmia after myocardial infarction by activating the AMPK signaling pathway and up-regulating the Cx43 levels (58). Therefore, there is no denying that the dual effects of SGLT2i on SGLT1 and SGLT2 are also beneficial to the heart (58). The success of the SOLOIST-WHF experiment (NCT03521934) also appears to presage a large potential for cardiac benefits with SGLT1 (59). Overall, the benefits of SGLT2 for AF can be explained by many other additional pathways of SGLT2i, instead of SGLT2, which is not distributed within the heart.

There is another interesting discovery in our research, which is the large difference in the results for AF vs. ventricular arrhythmias. We found that SGLT2i is associated with AF benefits rather than ventricular arrhythmias, and this result was consistent with the conclusion of Li et al. (11). Instead, Azam et al. found in an ischemia-reperfusion (I/R) isolated model that empagliflozin treatment significantly reduced the onset of VF induced by direct electrical stimulation compared to controls (60). In a *post-hoc* analysis of DAPA-HF, the addition of dapagliflozin to conventional therapy in HFrEF (heart failure with reduced ejection fraction) patients was found to reduce the risk of any severe ventricular arrhythmia, cardiac arrest, or sudden death (61). This discrepancy may be caused for the following reasons: Firstly, due to the insufficient number of these events (VF occurred in only one case in the EMPEROR-Reduced trial), it is highly likely to bias the experimental conclusions. Secondly, many studies have shown that the effect of SGLT2i on heart rhythm is closely related to the length of follow-up, which could be one of the reasons for the inconsistency (62). At present, there is still no research to discuss the association between SGLT2i and ventricular arrhythmias, but we still believe positively in the benefits of SGLT2i for ventricular arrhythmias. Whether SGLT2i is related to the reduction of ventricular arrhythmias will become one of our future research directions. To be sure, well-designed prospective trials are needed to prove this hypothesis. At a minimum, arrhythmic events should be included as a primary endpoint.

### Limitations

There are several limitations to this meta-analysis. Firstly, AF was not a pre-specified or adjudicated endpoint in any of these studies, which may have caused our conclusion to deviate from reality. However, since the studies we included were all with low overall deviation risk, the results and data were very clear, and largely consistent associations were observed. In addition, previous studies also adopted the method of taking adverse events as results (10, 63). To the authors' knowledge, none of the published trials of SGLT2i included the occurrence of AF or other arrhythmic events as an endpoint event. Therefore, we expect that the studies aiming to analyze the association of SGLT2i with AF or other arrhythmic events will provide more reliable data for our subsequent analyses. Secondly, two large-sample RCTs contributed 90% of the subjects in our analysis (7, 20). However, these two RCTs provide a high level of evidence, a large population size, and a rigorous experimental process. This does not affect the reliability of our results. Thirdly, EMPEROR-Preserved is a state-of-the-art, high-quality RCT. However, the results of this experiment have not been fully published, and we cannot find the results we are interested in. Therefore, we didn't include this experiment. Fourthly, most of the trials did not provide baseline level data for patients, such as the baseline prevalence of T2DM. Therefore, we could not perform subgroup analyses based on the presence or absence of diabetes to confirm the AF benefits of SGLT2i to HF patients without T2DM.

## Conclusions

In conclusion, our meta-analysis found that the use of SGLT2i in HF patients with and without T2DM was associated with a 37% reduction in the risk of AF events. On the contrary, it wasn't connected with the risk of AFL, AF/AFL, VF, VT, VF/VT, and bradycardia. Our study confirms the AF benefits of SGLT2i (dapagliflozin and empagliflozin) and provides strong evidence for its use in HF patients to reduce the risk of AF.

## Data Availability Statement

The original contributions presented in the study are included in the article/Supplementary Material, further inquiries can be directed to the corresponding author.

## Author Contributions

ZY and HZ performed the study design implementation and feasibility analysis, statistical processing, analysis and interpretation of results, wrote the paper, data collection and organization, revised, and reviewed the paper. ZG was responsible for the overall article and supervised the management. All authors reviewed, revised, and approved the manuscript. All authors had access to all data in the study and were responsible for submitting it for publication. All authors contributed to the article and approved the submitted version.

## Funding

We would like to acknowledge funding from the National Natural Science Foundation of China (No. 81673955), the Provincial Key Research and Development Project of Hunan (No. 2022SK2012), and the Provincial Natural Science Foundation Project of Hunan (No. 2021JJ30495).

## Conflict of Interest

The authors declare that the research was conducted in the absence of any commercial or financial relationships that could be construed as a potential conflict of interest.

## Publisher's Note

All claims expressed in this article are solely those of the authors and do not necessarily represent those of their affiliated organizations, or those of the publisher, the editors and the reviewers. Any product that may be evaluated in this article, or claim that may be made by its manufacturer, is not guaranteed or endorsed by the publisher.

## Abbreviations

HF: heart failure
AF: atrial fibrillation
AFL: atrial flutter
VF: ventricular fibrillation
VT: ventricular tachycardia
SGLT-2i: sodium -glucose cotransporter 2 inhibitors
T2DM: type 2 diabetes mellitus
RCT: randomized controlled trial
HFrEF: heart failure with reduced ejection fraction
HFpEF: heart failure with preserved ejection fraction
ROS: reactive oxygen species
MI: mitral insufficiency
CI: confidence interval
OR: odds ratio
NCX: Na^+^/Ca^2+^ exchanger
CaMKII: Ca/calmodulin-dependent kinase II
NHE1: Na^+^/H^+^ exchanger 1
ATP: adenosine triphosphate
PGC-1α: peroxisome proliferator-activated receptor γ coactivator-1 alpha
β-HA: β-hydroxybutyric acid.
